# Pioneer thought leader and scientist: Dr. Marcia G. Ory and her contributions to aging and public health across the life course

**DOI:** 10.3389/fpubh.2022.987137

**Published:** 2022-08-25

**Authors:** Deborah Vollmer Dahlke

**Affiliations:** ^1^DVD Associates, Austin, TX, United States; ^2^Texas A&M Center for Population Health and Aging, College Station, TX, United States

**Keywords:** female, mentor, public health, thought leader, aging, life course

It is not often you remember the first time you laid eyes on a person. For me, meeting Marcia at a conference on aging and cancer in 2010 was one of those life-changing events. Marcia is Marcia G. Ory, Ph.D., MPH, a Regents and Distinguished Professor of Environmental and Occupational Health at Texas A&M's School of Public Health. Marcia's contributions to research and health sciences are many, as is the national and international recognition she continues to receive, demonstrating her stature and leadership. Leading collaborative research teams, she has published prolifically (10 edited books, 45 book chapters, 20 special issues in professional journals, and approximately 470 peer-reviewed articles), cited in about 30,000 scholarly products, and been a key investigator in grants totaling more than $50 million. Just this year (2022), she made the Stanford University List of the World's Top 2% Scientists (1). She is listed as 88th in the United States and 177th globally among the Top 1,000 Scientists in Social Sciences and Humanities. Standing with her on this list are thought leaders such as Drs. Noam Chomsky, Talcott Parsons, and Everett Rogers (2).

Marcia's thought leadership in aging and public health is founded in her most cited articles including work on frailty and injuries, effects of exercise on falling among older adults, and chronic disease self-management (3–5). She has been recognized as a leading public health researcher by the American Public Health Association Aging and Public Health Section for her many contributions. These include the 2005 Archstone Foundation Excellence in Program Innovation Award (Honorable Mention), 2010 Philip G. Weiler Leadership in Aging Award, and 2014 Lifetime Achievement Award. She was also a finalist for the 2018 ASPPH Harrison C. Spencer Outstanding Community Service Award for her research and service in South Texas. Other notable accomplishments include Fellow status in several Professional Organizations: the Texas Public Health Association, the American Academy of Health Behavior (AAHB), the Gerontological Society of America (GSA), and the Society for Behavioral Medicine (SBM). She has also received the AAHB 2016 Research Laureate, GSA 2001 Award for Excellence in Applied Gerontology and 2007 Distinguished Mentorship in Gerontology Award, 2018 HealthCare Leadership Council Redefining Health in America Award, and 2019 Texas Department of Health and Human Services Innovators in Aging Award.

When I met Marcia, I was looking for a doctoral program in public health and was considering the University of Texas (UT) in Austin, where we lived, or maybe UT San Antonio, where I worked at the Cancer Therapy and Research Center and knew professors at the school. Marcia suggested I consider the Texas A&M School of Public Health in College Station, TX. It took just one visit to the school, the one where I had my interview with the recruitment committee, to lock me into driving 5 h round trip twice weekly from Austin to College Station to attend classes. I don't think I ever took a class with Marcia, but she certainly took me under her wing as an advisor and mentor and made sure that my experience at Texas A&M was everything it could be and that whatever I wanted my doctoral education to be was likely possible.

The classical idea of a mentor/mentee relationship is one of a more experienced, usually older, adult who supports and encourages a less experienced person in their professional endeavors. I was a well-experienced 61-year-old when I started my doctoral program at Texas A&M—the same age as Marcia. Our experiences were vastly different. In my 12 years of consulting at McKinsey & Company and Deloitte and Touche, LLC, and over 20 years in technology entrepreneurship and cancer care, I had only a few mentors—all of them male. Academia was a foreign land to me, and I was lucky to have Marcia as my guide. As a distinctly non-traditional student, my chosen subject area of adolescent and young adult (AYA) cancer survivorship was not, at least at that time, a traditional area of public health research, nor was my interest in building mobile applications to help AYAs live healthier lives. Marcia was relentless in finding ways for me to accomplish my vision, and she soon shared that vision. As an academic leader and mentor, Marcia just didn't lead the way; she helped me find and open the paths I needed to accomplish my goals. Beyond being a mentor, as she was and continues to be for many master's and doctoral students at Texas A&M's School of Public Health, Marcia is a transformational leader.

In the summer after my first semester as a doctoral student, Marcia approached me with an offer. She had grant funds related to a healthy living project that needed to be expended by the following August. Did I have any ideas? As we embarked on building the Healthy Survivorship mobile application for AYAs, I had no idea we were breaking new ground at the University. In my prior world of technology entrepreneurship, you built and tested new technology, but there were seldom any visible barriers or hurdles that weren't related to the technology or one's skill set. Academia was a different world, as I came to understand when we were summoned to meet with the Texas A&M University System's legal department. Marcia suggested I might want to wear a suit. The University attorneys were appropriately concerned with several things in the design, ownership, privacy, and disclaimers for the mobile application. Soon, we learned that our small effort was the first mobile application being built and released for the Texas A&M System. It was in this experience that I recognized Marcia's unique and powerful skill in creative problem-solving. Together, we developed ways to insulate the University from possible harm from the mobile app or legal threats from its potential future users. We also recognized the importance of having apps be theoretically based (6). Marcia's demonstration of creative problem-solving, negotiation, and communication skills were priceless lessons she often demonstrated throughout my doctoral journey.

Marcia is no stranger to the need to foster creative organizational change through direct and inspirational thought leadership. She has an outstanding research career starting with the 20 years she spent engaging in and promoting behavioral sciences research at the National Institute on Aging^1^. Her early research was transformational in defining the complex interactions among aging, health, and behavior processes. Marcia joined the Texas A&M University faculty in 2001 with a vision to create a robust aging and public health research agenda. As founding director of the Center for Population Health and Aging, she is known for her work on identifying risk factors for morbidity and mortality across the life course and for implementation and dissemination research on designing and evaluating multifaceted behavioral, social, technological, and environmental interventions. In a male-dominated environment, Marcia rose to become the only regents and distinguished professor at the School of Public Health, and one of the few across the entire Texas A&M University System with this combined distinction, an especially rare accomplishment for female scientists.

In 2016, Marcia helped transition the Texas A&M Program on Healthy Aging to an established Board of Regents Center for Population Health and Aging—the Center cut across all of the University campuses statewide and involved researchers from across the University System including Texas A&M's Rangel College of Pharmacy, Department of Biomedical Engineering, and College of Veterinary Medicine in addition to the School of Public Health (7). In 2018, she was named the Associate Vice President for Strategic Partnerships and Initiatives at Texas A&M and helped spearhead the Healthy South Texas Initiative which was designed to reduce chronic and infectious diseases prevalent in low-resourced communities (8, 9). Most recently, she has focused on furthering translational behavioral science with a passion for putting research into practice that can change the lives of older individuals, their families, and communities (10–12).

Marcia continues to propel the spread of evidence-based programs for older adults by identifying how they can meet the triple health care aims of better health, better healthcare, and better value. Her research, based on sound public health concepts and methodologies, continues to challenge aging stereotypes, including our collaborative work on new technology use by both aging adults and their caregivers (13, 14). Marcia didn't coin the term “healthy aging,” but she has undoubtedly helped to reconceptualize it as the new normal (15). In addition, Marcia continues her leadership to bring evidence-based programs to rural and underserved populations to reduce health disparities in access to care, including access to clinical trials and healthcare delivery through broadband access (16).

Throughout her career at Texas A&M, Marcia has mentored many students and junior faculty as recognized by receipt of the 2021 TAMU Women's Faculty Network Outstanding Mentoring Award. These mentees continue as researchers and leaders locally, nationally and globally in public health, health policy, and non-profit health organizations. The community of practice fostered by Marcia will continue with the vision and missions she inspired to change perceptions of aging to those of healthy aging, to find ways to creatively address economic, age, race/ethnicity, and geographic barriers to health and healthy lifestyles across the life course.

## Author contributions

The author confirms being the sole contributor of this work and has approved it for publication.

## Conflict of interest

Author DVD was employed by DVD Associates.

## Publisher's note

All claims expressed in this article are solely those of the authors and do not necessarily represent those of their affiliated organizations, or those of the publisher, the editors and the reviewers. Any product that may be evaluated in this article, or claim that may be made by its manufacturer, is not guaranteed or endorsed by the publisher.

## Footnotes

^1^Available online at: https://en.wikipedia.org/wiki/Marcia_G._Ory [Retrieved May 31, 2022].
